# Supplementary material for: Collaborative intelligence in AI: Evaluating the performance of a council of AIs on the USMLE
Source: PLOS Digit Health. 2025 Oct 9;4(10):e0000787. doi: 10.1371/journal.pdig.0000787 (PMC12510544; doi:10.1371/journal.pdig.0000787)
Supplement: S2 File — (ZIP) [file pdig.0000787.s002.zip › S2 Data Raw data files containing unprocessed question inputs and LLM outputs including transcripts of deliberations/STEP3_Question-74-1_Type-MC-NJ.docx]

**Facilitator** **[Round 1]:** A 45-year-old woman comes to the office because of a 3-day history of nasal congestion, headache, sore throat, and general malaise. She has developed a cough during the past 24 hours that is mildly productive of clear sputum. Except for occasional seasonal allergies, she has no previous history of medical problems. She takes no medications on a regular basis but during the past 3 days she has been taking an over-the-counter oral cold medication. She follows a strict low-fat vegetarian diet and she drinks three to four glasses of wine per week. She jogs 3 miles a day when she is not sick. Vital signs today are temperature 37.5°C (99.5°F), pulse 90/min, and blood pressure 140/94 mm Hg. Height is 170 cm (5 ft 7 in); weight is 54 kg (120 lb); BMI is 19 kg/m². On physical examination she appears slightly anxious. Nasal mucosa is erythematous and swollen, and sinuses are nontender. Posterior pharynx is also erythematous but there are no exudates. Lungs are clear on auscultation and cardiac examination is normal. Repeat blood pressure measurement at the end of the examination is 142/96 mm Hg in the left arm and 138/94 mm Hg in the right arm. Which of the following is the most likely cause of her elevated blood pressure? (A) Acute viral illness (B) Chronic sinusitis (C) Essential hypertension (D) Her alcohol intake (E) Over-the-counter cold medication

**AI_B:** RESTATING THE QUESTION: This middle-aged woman has been displaying symptoms of an upper respiratory infection, such as nasal congestion, a sore throat, and a cough productive of clear sputum, for the last few days. Despite having no previous medical history, her blood pressure readings have been consistently high. The cause of this newfound hypertension is what we are looking to determine. The options provided range from her current assumed viral illness, long-term sinusitis, essential (or primary) hypertension, her weekly alcohol intake, or the cold medication she's been taking. PLANNING: Initially, one could assume that the immediate viral illness or usage of cold medication may be the cause of her elevated blood pressure. Acute illnesses can cause stress responses in the body, resulting in high blood pressure. Some over-the-counter cold medications contain substances that could raise blood pressure. However, her relatively modest alcohol intake and the possibility of her having undiagnosed essential hypertension can't be overlooked. Accordingly, an alternate assumption would be that the essential hypertension, her alcohol intake, or even a persistent condition like chronic sinusitis could be responsible for her high blood pressure. CLINICAL REASONING: Essential hypertension usually develops over time and is often asymptomatic until discovered during a routine check-up or when complications develop. With the patient's age being 45, it is possible she could have essential hypertension. However, without a history of high blood pressure readings, labelling it as essential hypertension might be precipitous. Chronic sinusitis can result in elevated blood pressure due to chronic pain or discomfort, but the patient's sinuses are reported as nontender, lowering the probability of this option. Moderate alcohol intake can contribute to high blood pressure, but the quantity this patient consumes seems unlikely to cause a significant elevation. Lastly, certain over-the-counter cold medications contain phenylephrine or pseudoephedrine, decongestants that can raise blood pressure. Given her recent onset of symptoms and usage of these medications, this appears as a plausible cause for the elevated blood pressure. CONCLUDING: Given this clinical scenario, an acute condition that directly increases blood pressure seems more likely than a chronic condition. Without a previous history of hypertension and taking into account her age and lifestyle habits, such as a low-fat diet and regular physical activity, essential hypertension becomes a less likely culprit. RESPONSE: Therefore, her elevated blood pressure is most likely due to the (E) Over-the-counter cold medication she has been taking.

**AI_E:** (1) RESTATING THE QUESTION: The question pertains to the possible cause of the patient's elevated blood pressure, given her medical history. Elevated blood pressure, also known as hypertension, occurs when the force of blood against the walls of blood vessels is consistently too high. Various conditions and lifestyle factors can contribute to this including certain medications, chronic diseases, and unhealthy dietary habits among others. (2) PLANNING: The assumptions here could start with the patient's history of seasonal allergies, intake of an over-the-counter oral cold medication, alcohol consumption, and her physical health condition which includes exercise and a vegetarian diet. There's also her current symptoms of a viral-like illness, her anxiety, and her physical state - erythematous and swollen nasal mucosa with a clear lung auscultation result. However, the leading assumption of interest here would be the over-the-counter cold medicine use for 3 days given that certain cold medications are known to cause a rise in blood pressure. (3) CLINICAL REASONING: Evaluating each assumption, while Acute viral illness (A) could cause a temporary increase in blood pressure due to an inflammatory response, her symptoms and physical findings are not typically associated with a significant rise in blood pressure. Chronic sinusitis (B) might cause pain and discomfort, but it's not typically associated with elevated blood pressure. The term Essential hypertension (C) refers to chronic high blood pressure with no identifiable cause and is often linked to genetics, age, or lifestyle. But given her history, nothing pointed directly to it. Alcohol intake (D) can indeed lead to elevated blood pressure, but three to four glasses of wine per week might not have a significant effect on blood pressure. However, Over-the-counter cold medication (E) can potentially elevate blood pressure. This is particularly true for preparations that contain decongestants such as pseudoephedrine, which may raise blood pressure. (4) CONCLUDING: Given her 3-day history of taking an over-the-counter cold medicine, the absence of any significant past medical issues or factors, and ruling out other options, it becomes arguable that her elevated blood pressure is most likely due to the Over-the-counter cold medication. (5) RESPONSE: Based on the current information provided and the clinical reasoning process outlined above, the most likely cause of this 45-year-old woman's elevated blood pressure is the Over-the-counter cold medication (E). It's generally advisable for hypertensive patients or people with a tendency for high blood pressure to avoid certain over-the-counter cold medications due to this potential side effect.

**AI_D:** RESTATING THE QUESTION: The query here revolves around a 45-year-old woman presenting with symptoms of a cold, which have been persisting for the last 3 days. Along with these symptoms, she's been taking over-the-counter cold medication during this period, and leads an active lifestyle and adheres to a low-fat vegetarian diet. She also drinks 3-4 glasses of wine weekly. Although her health is generally good, currently she is noted to have escalated blood pressure. The investigation seeks to identify the most likely cause of this high blood pressure, which could be due to her acute viral illness, chronic sinusitis, a condition known as essential hypertension, alcohol intake or the over-the-counter cold medication she's been using. PLANNING: In terms of assumptions, there's the general idea that her high blood pressure is related to her current illness state or a chronic condition. By focusing on these assumptions, we can also think of other possibilities that may be influencing her blood pressure. This could include her recent medication use, lifestyle choices, or even a combination of these elements. Reviewing the assumptions and judging them in a light of clinical priorities points towards the over-the-counter cold medication as one of the primary things to consider, due to its immediate timing and potential effects on blood pressure. CLINICAL REASONING: The patient's symptoms of nasal congestion, headache, sore throat, general malaise and productive cough are suggestive of an acute viral respiratory infection. However, elevated blood pressure is not typically associated with these types of viral illnesses. Chronic sinusitis could cause similar symptoms with this patient's, but like in the case of acute viral illnesses, would not be the probable cause of elevated blood pressure. Essential hypertension generally implies a chronic, primary elevated blood pressure without known cause. It is less likely considering the absence of prior blood pressure problems indicated by her medical history. Although alcohol can contribute to hypertension with high intakes, the amount consumed by this patient probably would not induce hypertension. Over-the-counter cold medications, on the other hand, often contain sympathomimetics like pseudoephedrine, which can cause marked elevation in blood pressure. Given the correlation between the timing of her symptoms and taking this medication, as well as the known effects of such drugs, they appear as a very potential cause for her elevated blood pressure. CONCLUDING: After careful consideration using a step by step approach involving restating the question, planning, and clinical reasoning, it appears that the most likely cause of this patients elevated blood pressure is the over the counter cold medication that she has been taking for her cold symptoms. This reinforces the need to monitor medication use, particularly over the counter options, due to potential side effects like the one mentioned here. RESPONSE: (E) Over-the-counter cold medication is the most likely cause of her elevated blood pressure.

**AI_C:** RESTATING THE QUESTION: The patient in question is a 45-year-old female who has recently been feeling unwell, with symptoms like nasal congestion, headache, sore throat, and general malaise. Over the past day, she has also developed a cough. Her past medical history is relatively clear, excluding seasonal allergies. During this bout of sickness, she's been taking over-the-counter cold medicine. Her lifestyle includes a low-fat vegetarian diet, moderate alcohol consumption, and regular exercise when she's feeling well. Upon physical examination, her nasal mucosa is inflamed and swollen, an observation that is also applicable to her pharynx but there is no exudate presence. Additionally, her lungs are clear, and her cardiac health appears to be normal. However, her blood pressure readings are elevated. The question is - what is the most likely cause for her high blood pressure? PLANNING: Assuming different factors to be responsible for the patient's elevated blood pressure includes: acute viral illness, chronic sinusitis, essential hypertension, her alcohol intake, and over-the-counter cold medication. Evaluating these factors, given her symptoms, it's plausible that an acute viral illness could be raising her blood pressure. Likewise, taking into consideration her use of over-the-counter cold medicine, this could also be a probable cause as certain cold medicines can cause a spike in blood pressure. CLINICAL REASONING: One might think acute viral illness can lead to systemic effects and, in some cases, cause elevated blood pressure, but in this instance, we should consider whether the virus causing her symptoms would likely lead to persistent hypertension; such outcome is generally unlikely. It's also worth noting that essential hypertension usually presents as a chronic condition, and given her healthy lifestyle, with no previous history of hypertension, this conclusion seems less likely. Her alcohol intake is moderate, not likely to be an immediate cause for her somewhat significantly elevated blood pressure. Coming to the over-the-counter cold medication, some of these medicines do contain elements such as pseudoephedrine, known to constrict blood vessels and potentially elevate blood pressure levels. CONCLUDING: Given the symptomatology, recent history of cold medication intake, and the absence of any previously recorded blood pressure problems, combined with the continuation of her elevated blood pressure over the course of the examination, it would be reasonable to associate the elevated blood pressure with the intake of the over-the-counter medication. This conclusion, however, does not exclude other causes and should be made with consideration of the full clinical picture - her symptoms, past medical history and lifestyle. RESPONSE: (E) Over-the-counter cold medication is the most likely cause of her elevated blood pressure.

**AI_A:** Restating the Question: A 45-year-old female who maintains good health habits and exercises regularly, has come for a checkup due to recent symptomatic disturbances. She has a history of nasal blockage, headaches, throat discomfort, and feelings of overall weakness for the past three days. In the last day, she has also developed a cough that brings forth transparent sputum. Except for seasonal allergies, her medical history is virtually absent of any chronic illnesses. She has been taking an over-the-counter cold medicine for the last three days. Despite her recent illness, she remains active and maintains a low-fat vegetarian diet. Her present vital signs marked an elevated blood pressure. Additionally, her nasal mucosa shows signs of inflammation. The question is, what could be causing her high blood pressure? Planning: Evaluation is required for each of these assumptions: 1) Is her elevated arterial pressure due to the acute viral infection? 2) Could her chronic sinus be responsible? 3) Is this a case of primary hypertension that has only been identified now because of her current symptomatic presentation? 4) Could her alcohol consumption be contributing to her increased blood pressure? 5) Finally, could her over-the-counter cold medication be causing her hypertension? Clinical Reasoning: Taking all symptoms into account, her presentation seems to suggest an acute viral upper respiratory infection rather than chronic sinusitis, which rules out option (B). Acute viral illnesses such as the common cold can sometimes cause transient elevations in blood pressure due to systemic inflammation, but these outbreaks are generally mild and unlikely to cause the level of hypertension seen in this case, thereby bringing option (A) into question. While her alcohol intake could theoretically contribute to hypertension, her consumption at three to four glasses per week is not typically enough to cause a significant elevation, meaning we should also doubt (D). Essential hypertension, or primary hypertension, could indeed be a cause. It's mostly seen in patients over the age of 50, and it often presents without any symptoms. However, the sudden and marked increase in blood pressure in this patient, who leads a healthy lifestyle and has never had high blood pressure previously, makes (C) a less likely solution. In contrast, many over-the-counter cold remedies contain sympathomimetic agents such as pseudoephedrine, which can lead to a significant elevation in blood pressure. Since the high blood pressure coincides with the symptoms of her infection and the use of cold medication, we can find a strong correlation there. Concluding: Given that many non-prescription cold remedies contain drugs designed to constrict blood vessels, this woman's high blood pressure is likely a side effect of the over-the-counter cold medicine she has been using. The fact that these symptoms overlap with those of her infection and her use of this medication makes a strong case for this assumption. Response: Therefore, the most likely cause of her elevated blood pressure is her over-the-counter cold medication (E).

**Facilitator** The consensus among the AI responses is that the most likely cause of the 45-year-old woman's elevated blood pressure is the over-the-counter cold medication she has been taking (E). This conclusion is reached through a process of elimination and clinical reasoning. While acute viral illness and chronic sinusitis could theoretically cause elevated blood pressure, these conditions are not typically associated with significant hypertension. Essential hypertension, a chronic condition with no identifiable cause, is also considered less likely due to the patient's healthy lifestyle and lack of previous blood pressure issues. Although alcohol can contribute to hypertension, the patient's moderate intake is not expected to cause a significant elevation in blood pressure. On the other hand, many over-the-counter cold medications contain substances such as pseudoephedrine, which are known to constrict blood vessels and potentially elevate blood pressure levels. Given the timing of her symptoms and the use of this medication, there is a strong correlation suggesting that the cold medication is the most probable cause of her elevated blood pressure. However, it's important to note that this conclusion does not exclude other potential causes and should be made with consideration of the full clinical picture, including her symptoms, past medical history, and lifestyle.
